# Engineering Ribosomes to Alleviate Abiotic Stress in Plants: A Perspective

**DOI:** 10.3390/plants11162097

**Published:** 2022-08-12

**Authors:** Leticia Dias-Fields, Katarzyna P. Adamala

**Affiliations:** Department of Genetics, Cell Biology, and Development, University of Minnesota, 6-160 Jackson Hall, 321 Church Street SE, Minneapolis, MN 55455, USA

**Keywords:** ribosome heterogeneity, plant ribosome, abiotic stress, synthetic biology, stress tolerance, genetic engineering, perspective

## Abstract

As the centerpiece of the biomass production process, ribosome activity is highly coordinated with environmental cues. Findings revealing ribosome subgroups responsive to adverse conditions suggest this tight coordination may be grounded in the induction of variant ribosome compositions and the differential translation outcomes they might produce. In this perspective, we go through the literature linking ribosome heterogeneity to plants’ abiotic stress response. Once unraveled, this crosstalk may serve as the foundation of novel strategies to custom cultivars tolerant to challenging environments without the yield penalty.

## 1. Introduction

Environmental stressors have a major negative impact on plants’ life cycle [1]. They prevent the crops from delivering 70% of their production potential. This means that the actual crop yield average corresponds to only 30% of what would be in the absence of environmental stress [2,3,4,5]. Considering not only the impossibility of having an environment free of abiotic disturbances but also the current aggravation due the global warming, the urgency for new tolerant cultivars becomes unquestionable. To reach this goal in a fast and efficient manner, approaches such as synthetic biology technologies should be made use of.

Synthetic biology proposes to go beyond genetic engineering by manipulating not only genes but whole metabolic systems and their regulatory pathways [6]. The ribosome has been a primer target of synthetic biology efforts [7], mainly due to its capability to synthesize protein at an extraordinary rate and accuracy [8]. So far, the efforts were mainly directed to use its workforce to add nonstandard amino acids to a protein or even translate another kind of polymers [9,10,11,12,13]. However, rising evidence of ribosome subpopulations responding to environment stress in plants have opened new harnessing possibilities [14,15,16,17,18].

The ribosome is the workhorse of translation machinery [19]. While it was initially understood as an invariant and passive organelle, comprehension about its role has gotten new colors in the last decades [20,21,22]. The differential expression of individual ribosome components that was detected in many organisms, including plants, led to the acknowledgment of ribosome heterogeneity [21,23,24,25,26,27,28,29]. Additionally, details stating that its activity is strongly linked with external signals have also been uncovered [30,31,32,33,34,35,36]. Now, efforts have been put into understanding better how the ribosome variety serves to fulfill the cell demands in a dynamic environment [37].

As immobile beings, plants rely only on internal processes to deal with environmental pressures [38,39]. Itdemands an efficient response system built on a finely tuned gene expression. Among all the levels of gene expression regulation, the translational one stood out as an important via to attenuate the cell apoptosis process triggered by abiotic stress [40]. Free of the energy and time costs charged by the de novo mRNA synthesis, the translational regulation can be faster and more dynamic [41,42]. In addition, the absence of alterations in mRNA levels allows a prompt recovery when the stress factors are removed or reduced [43,44].

Cells’ primary demand in plants going through adversities is to safeguard energy [45]. They do that by suppressing energetically consumptive processes such as protein synthesis [41]. This way, the energy before invested in growth is redirected to physiological adaptations that help the plant thrive through harsh conditions [46]. Although quite efficient to guarantee survival, the resulting stress tolerance is achieved at productivity expenses [47].

In the world’s actual scenario, it presents a huge challenge to farmers and scientists. The rapid expansion of population requires an increasing agricultural output, but global climate deterioration does not favor it. Therefore, the food security of the next generations depends on the creation of cultivars that conjugate two conflicting traits under adverse conditions, tolerance, and high yield, which is an ambitious task that requires creative strategies to be accomplished.

**Figure 1:**
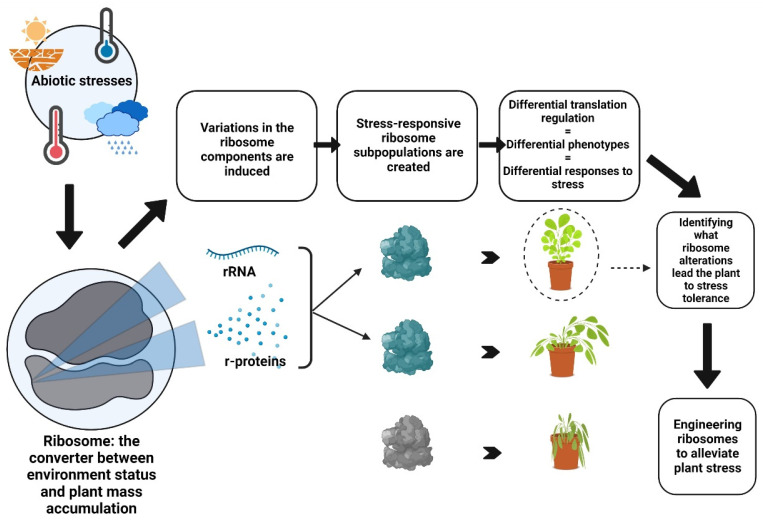
The crosstalk between ribosome heterogeneity and abiotic stress response in plants (Figure created with BioRender.com, accessed on 25 July 2022).

The tight coupling with external stimuli and its pivotal regulatory role in cellular proliferation place the ribosome as a converter between environment status and mass accumulation. Once understood and decoded, this capability can be engineered to construct ribosome variants that may set an atypical growth pace in response to abiotic stress signaling. This establishes thus the basis for the creation of stress-tolerant cultivars which are also productive under hostile environments (Figure 1).

Although quite promising, harnessing ribosomes to promote plant tolerance demands a deeper knowledge of plant ribosomes heterogeneity and its association with abiotic stress response. In this perspective, we review our current understanding about it. The goal here is to outline what is already known and discuss what is still to be caught in order to make this approach feasible.

## 2. Ribosome Heterogeneity: A Platform for Custom Stress-Responsive Ribosomes

The noncanonical compositions of translation machinery classify as ribosome heterogeneity [48]. It includes any level of variations in ribosomal ribonucleic acid (rRNA) or ribosomal proteins (RP) [24,49,50]. Identifying what alterations make a ribosome responsive to stress and how they drive the translatome to meet the plant physiological demands in a disturbed environment is the foundation that the prospect of customizing ribosomes to increase plant resilience is built on (Figure 1).

The way ribosome heterogeneity works for gene regulation is still a controversial matter. Two contrasting explanations were proposed: the “insufficiency” and the “specialization” hypotheses [48]. The “insufficiency hypothesis” defends that structural changes produce inoperative ribosomes [49]. A deficit of functional ribosomes is thus established, and the translation preference becomes determined for the majority by the mRNA sensitivity to ribosome concentration. As reviewed by Ferretti and Karbstein (2019) [51], the extent to which the expression of a gene is affected by the ribosome availability is defined by its 5′ untranslated region (UTR) content and size.

UTR elements as Kozak sequences and upstream open reading frame (uORF) play a great part in the translation efficiency [52]. The Kozak motif is the consensus distribution of nucleotides surrounding the translation initiation site (TIS) [53]. The more conserved it is, the stronger the signal of TIS recognition is transmitted and the more intense the ribosome recruitment is [54]. That is why genes carrying an optimal Kozak motif in their UTR are most likely to be successfully translated even under ribosome low availability [55]. In plants, Gupta et al. (2016) [56] identified GCNAUGGC, AANAUGGC, and GCNAUGGC as Kozak consensus sequences for monocots, dicots, and plants in general, respectively.

The uORFs are found in 24–30% of the total plant mRNAs [57]. Their presence tends to attenuate the rate of protein synthesis, which attaches to them a regulatory attribute already reported to be involved in the plant response to abiotic stress [58,59,60,61,62,63,64]. They typically cause the stalling, or simply the dissociation, of ribosomes. In these cases, in order to have the main ORFs (mORFs) expressed, the translation needs to be reinitiated [62,65]. It requires an abundance of functional ribosomes and a UTR big enough to allow them to bound, reacquire the translation initiation factors, and then resume the scanning for the main start codon [66]. Accordingly, genes with long 5′ UTR as well as the ones carrying uORF or weak Kozak sequences are more dependent on the ribosome concentration to be translated [51,67]. In Srivastava et al. (2018) [57], the function specificity and UTR length data of plant genes were analyzed, crossed, and organized in categories. The results showed that in Arabidopsis and rice, the genes involved in stress response bear short (1–500 bp) and medium (1001–2000 bp) UTR, respectively, suggesting that their translation products are much more likely to be prioritized in the case of ribosome numbers decreasing.

In the specialization hypothesis though, the ribosomes are the ones that deal out the cards of translation preference. Unlike the insufficiency hypothesis that sees the ribosome structural alterations as a source of functional corruption, the specialization one states they are a source of functional diversity which can be manifested by deviant translational fidelity, speed, and/or mRNA selectivity [21,23].

Both classes of heterogeneous ribosomes, nonfunctional and specialized, exert regulation even through different action modes. It is not simple to distinguish which of the phenomena is responsible for each certain translation outcome [51]. In plants, the phenotypes derived from knockout/knockdown of specific RP mutation generally share some developmental anomalies such as impaired growth, reduced cell proliferation, and increased nuclear ploidy in leaf cells [49,68]. The high similarity between these phenotypes and those resulting from general ribosomal depletion leaves room for questioning whether the regulatory activity associated with specialized ribosomes is actually coming from a scarcity of functional ribosomes rather than from a distinct performance.

In fact, the integration of the two hypotheses is the more reasonable explanation of how ribosome heterogeneity modulates the translation. Regulation is an extremely complex process that involves intricate pathways triggered by a profusion of signals of different intensities and duration. It is expected then, as an important piece of this movement, that the ribosome constitution and activity be impacted in as many ways as diverse as the cell signaling. All these things considered, it can be assumed that either specialized or nonfunctional heterogeneous ribosomes work together to line up the proteome with the cell’s demands that are constantly changing.

## 3. The Plant Ribosome and Its Vocation to Stress Response Regulation

The ribosomes are large cellular complex products of RNA and protein association. Two moieties, one large (60S) and one small (40S), compose the eukaryotic ribosome. The large subunit consists of 25S/28S, 5.8S, and 5S ribosomal RNA conjugated with 47 proteins. The small subunit is the set of 18S RNA plus 33 proteins [69]. This elaborated structure opens vast possibilities to merge component variants, thus creating heterogeneity that may lead to singular translational activities.

Below, some specific features of plant ribosomes and their biogenesis are listed, and the evidence associating them with abiotic stress response is addressed. Their convergence point is that they all render resources to convert external signals into ribosome variability through changes in the rRNA synthesis, rRNA modifications, and/or protein content.

### 3.1. Ribosomal Proteins Heterogeneity: Variety to Face Adversity

Each RP that compounds the plant ribosome is codified by multiple genes which are members of several small families [70]. In their analysis, Carroll et al. (2008) [71] found that in Arabidopsis, the 33 RPs from the small subunit and the 48 ones from the large subunit are codified by 102 and 146 genes, respectively, averaging three predicted genes for each RP type. Even though the multigenic nature of RPs is commonly found in eukaryotes, most of the gene copies are pseudogenes [72,73,74]. The plants, however, are an exception to this pattern by having most of the RPs’ gene copies expressed and functional [70,75].

The gene sub-/neo-functionalization is promoted by polyploidization, the multiplication of whole chromosome sets [76,77,78]. Polyploidization is a widespread and recurring phenomenon in the plant kingdom that might be responsible for taking the plant RPs variability to this further level [68,79,80].

Initially seen only as the cement units that kept the rRNA together, shaping the ribosome, the RPs’ biological roles on and even off their contribution to translation have been acknowledged [81]. As they attach to or release from the ribosome subunits, the RPs confer not only new structural but mainly new functional conformations to the ribosome [82]. As reviewed by Byrne (2009) and Horiguchi et al. (2012) [49,68], experiments with RP-defective mutants revealed that the alterations in the number of specific RP families and/or paralogs, an event known as substoichiometry, produced abnormal developmental phenotypes in plants. Proteomic examinations in plants submitted to abiotic stress were also performed. They detected that RPs and ribosome biogenesis factors are among the major protein groups that are differentially abundant between stress-susceptible and stress-tolerant plant genotypes [83].

Although ribosome assembly is highly coordinated with cellular needs, Martinez-Seidel et al. (2020) [48] brought attention to the importance of considering the ribosome turnover to realistically assess the impact of RP changes in the plant response to environmental stress. In Arabidopsis, the mean half-life of RPs is 3–4 days [84]. Meaning that the de novo synthesis of substoichiometric ribosome subfamilies responsive to stress may not happen immediately after the external signal reception. Another possibility raised by the same authors is that alterations/replacements may also occur only to the proteins located on the ribosome surface. This way, once the ribosome core is preserved and reused, the generation of stress-specialized ribosome groups would be faster.

### 3.2. Plant Ribosome Stress Response: Finer, as It Should Be

Critical to the cell cycle progression, the ribosome biogenesis consumes 80% of an active eukaryotic cell’s energy and is highly sensitive to external stimuli [85,86]. The ribosome stress, also called nucleolar stress, happens when perturbations in the nucleolar morphology and/or function compromise the ribosomal biogenesis [87]. These perturbations can be created by a plethora of stress conditions such as changes in temperature and energy status, hypoxia, nutrient starvation, DNA damage, and UV light, among others [88,89,90].

In animals, the best-characterized response pathway to the ribosome stress is led by the p53 transcription factor. Known as the guardian of the genome due to its important role in cell cycle control and DNA repair [91], p53 is suppressed by MDM2 in unstressed cells. Upon ribosome stress, however, RPs are released from the nucleolus into the nucleoplasm, causing MDM2 inhibition and consequently p53 accumulation. Once stable, p53 induces the expression of a cohort of genes that are involved in cell cycle arrest, senescence, and/or apoptosis setting; this way, the cell responds to ribosome stress [92].

The p53 or MDM2 homologs are not found in plants. However, studies have demonstrated there are multiple proteins playing a very similar role in plant cells. Some of these proteins belong to the NAC family. NAC is a plant-specific transcription factor family largely involved in stress response [93,94,95,96,97]. As reviewed by Obayashi and Sugiyama (2018) [98], NAC transcriptions factors ANAC008, ANAC002, ANAC053, and ANAC082 are key mediators in Arabidopsis response to DNA damage, oxidative stress, and ribosome biogenesis disruptions, respectively. The same mechanisms are governed by p53 in animal cells.

The large number of factors in the plant cell performing the same function that is accomplished by only one protein in the animal cell may infer a finer level of response to nucleolar stress in plants. It corroborates with the several pieces of evidence presenting the plant nucleolus as a stress sensor by eliciting and modulating pathway response to drought, salinity, temperature variation, and others (reviewed by Kalinina et al. (2018) [90]).

### 3.3. Plant Nucleolar Vacuole: A Cavity Stuffed with Ribosome Heterogeneity

Plant nucleolus contains a structure that distinguishes it from the other ones. It is the nucleolar cavity, a vacuole in the center of the nucleolus whose function is still little known [90,99]. The nucleolar cavity has been indicated as a place of storage and temporal sequestration for mainly small nucleolar (snoRNA), among other biochemical factors such as spliceosomal small nuclear RNAs (snRNAs) and elements of the ubiquitin–proteasome system [100,101,102,103,104].

The snoRNAs have pivotal participation in the rRNA production by marking rRNA molecules to be modified [105]. The modifications are changes in the chemical composition of ribonucleic acid. They are part of a process called maturation which comprises a series of processing steps that lead to the releasing of individual mature rRNA strands from their precursor form, one long polycistronic molecule [106].

The effects of modifications are not limited to the chemical arrangement of rRNA molecules though. Usually clustered in functional rRNA regions as the binding sites for tRNA, the modifications apply a selection pressure on the other translation machinery components [105]. Translation factors, RPs, and tRNA that associate with rRNA may be compatible only with specific sets of rRNA modifications. It can establish a myriad of rRNA-interactor combinations which, in turn, may diversify the translation steering [107].

Studies have been showing that variations in the rRNA modification patterns are responsive to environmental stimuli in eukaryotic cells [108,109,110,111]. This shed new light on the putative function of the plant nucleolar cavity. Once it bears snoRNA, the guiders of rRNA modifications, the nucleolar cavity may perform as a bank of ribosome potential heterogeneity and consequently a booster of plant responsiveness to the environment.

### 3.4. TOR and SnRKs: The Connectors of Stress Signaling and Ribosome Biogenesis Regulation

Target of Rapamycin (TOR) is a protein kinase that works as a hub sensor and metabolism programmer in eukaryotic cells [112,113]. It catalyzes numerous processes according to the cellular nutrient status and environmental conditions [114]. Firstly found in yeast and animals [115,116,117,118], TOR has been a subject of great interest in plant research after findings placed it as a key coordinator of ribosome biogenesis and cellular adjustments to abiotic cues also in Arabidopsis [119,120,121,122,123,124]. Unable to move away from unfavorable environments, the plants demand an exquisite synchronization between these two processes. In order to survive, they must be extremely receptive to the signals from outside and efficient in converting them to cell growth adaptations. The data published so far point to TOR and sucrose-non-fermenting-1-related protein kinases (SnRKs) as the ones that drive plants toward this achievement [125].

The TOR active engagement in the regulation of plant ribosome biogenesis has been attested by plenty of studies. Ren et al. (2011, 2013) [122,126] and Kim et al. (2014) [123] showed in Arabidopsis that rRNA synthesis is regulated by TOR, as previously demonstrated in yeast and mammals [127,128]. The TOR control over the expression of plant RPs was reported by Xiong et al. (2013) [129], Dobrenel et al. (2016) [130], and Bakshi et al. (2020) [131]. Furthermore, TOR involvement in the coordination between nucleotide biosynthesis and the cell demands for ribosome biogenesis was supported by data from Busche et al. (2021) [132].

Under challenging environmental circumstances, TOR works together with SnRKs transducing the induced signaling and modulating the plant response [125]. The SnRKs are the plant components of the SNF1-type kinases family, which also comprises AMPK and SNF1 itself, which are mammalian and yeast proteins, respectively [133]. They are divided into three subgroups—SnRK1, SnRK2, and SnRK3 [133]—totaling 38 members [134]. Groups 2 and 3 do not have animal or fungi correspondents [135].

The TOR-SnRK1 is a widely accepted pathway to explain the regulation of energy homeostasis in plant cells. Essentially, TOR and SnRK1 work closely to activate/inhibit energy-consuming (anabolic) or energy-releasing (catabolic) reactions in a “yin–yang” dynamic guided by cell energy status and phytohormones such as abscisic acid (ABA), the chief mediator of plant responses against environmental pressures [136,137,138]. Even though some research results have presented discrepancies from this model, as reviewed by [125], it is commonly acknowledged that TOR, activated by high sugar availability and low ABA levels, promotes anabolic routes such as ribosome biogenesis and protein synthesis [113,139,140,141]. SnRK1, on the other hand, is activated by the opposite conditions and triggers the catabolic pathways as autophagy, the degradation and recycling of damaged cellular components after a stress episode [142,143,144].

Regarding SnRK2 and SnRK3, extensive research has made clear they play a significant part in the ABA signal pathway [134,145,146,147,148,149,150,151,152,153]. As plant-exclusive members of SnRKs kinases, their performance can be understood as an evolutionary level up in plant responsiveness to external conditions [135,154].

The accumulating data demonstrate how plant ribosome activity is firmly coordinated with environmental factors through TOR and SnRKs orchestration. As a high energy demanding process, the ribosome biogenesis is under a constant regulation to help plant cells maintain the balance between energy storage and expenditure [155]. This regulation becomes even tighter under limiting conditions once the success of plant acclimation, and hence survival, is directly related to its ability to manage energy consumption [156].

## 4. Ribosome Heterogeneity and Abiotic Stress Regulation in Plants: What Is Already Known?

While much remains to be discovered, valuable data linking plant ribosome variability to environmental stress have been uncovered. Kawasaki et al. (2001) [18], Moin et al. (2016, 2017) [157,158], and Saha et al. (2017) [16] showed that ribosomal proteins are differentially expressed in rice submitted to osmotic and ionic stresses. Moin et al. (2017) [158] went further and also validated the influence of an RP gene, RPL23A, on rice’s capability to convert water into biomass. Rice plants overexpressing RPL23A performed better than the control ones when exposed to simulated drought and salinity conditions by presenting an increase in fresh weight, root length, and proline and chlorophyll contents.

The expression of RP genes was also reported to be induced by low temperatures in *Brassica napus* [159], soybean [14], rice [160], barley [161], and Arabidopsis [162], demonstrating that RP can be considered as potential targets for manipulation of tolerance in multiple crops. Moreover, Martinez-Seidel et al. (2021a, 2021b) [161,162] observed in barley and Arabidopsis, respectively, that significant changes in the relative amount of specific RP paralogs were triggered by cold acclimation, which could imply the generation of cold-induced ribosomes characterized by substoichiometric proteome composition. The ribosome regions where these alternative proteoforms are located and their effect on the translation flow were investigated by Martinez-Seidel et al. (2021b) [162]. The results showed that polypeptide exit tunnel (PET), P-stalk, and head portions of ribosome have their functionality limited by the structural remodeling provoked by cold stimulus.

The transcriptome analysis carried out by Garcia-Molina et al. (2020) [163] identified that translational components, especially ribosome proteins, are largely represented among the hubs found in the network of genes expressed in Arabidopsis plants under high light, heat, and cold stress. Hubs are the most highly connected genes in the co-expression network. They occupy a central role in signaling transduction by promoting the interaction between gene expression modules and hence the overlapping of stress response pathways [164].

Certainly, the structural proteins are the most documented source of heterogeneity in plant ribosomes so far, but they are not the only ones. Although the evidence of other sorts of variability are still short, some inferences can be made from the study of translation factors. As mediators of ribosome biogenesis and assembly, the translation factor’s responsiveness to the environment signals may reflect on ribosome diverse configurations. This idea is strengthened by the fact that in plants, the ribosome biogenesis count on plant-exclusive translation factors [165] and the rRNA processing is secured by alternative routes [68,166], suggesting that plant ribosomes may present a vast and specific repertoire of heterogeneity.

Arabidopsis REI1-LIKE proteins (REIL1 and REIL2), for instance, are ribosomal biogenesis factors that act in the rRNA processing. It turns out that they are also heavily involved with the plant response to cold stress [167,168,169]. The REIL proteins are required to keep the rRNA maturation process on even under low temperatures, thus preventing a severe growth downturn and stimulating cold tolerance in plants [170].

Another ribosomal biogenesis factor reported as responsive to stress is NOG1, nucleolar GTP-binding protein 1. This small GTPase is essential to the rRNA maturation [171]. Its silencing caused the over-accumulation of pre-rRNA processing intermediates and a subsequent decrease in mature rRNAs in *Nicotiana Benthamiana* [172]. In Arabidopsis, Lee et al. (2017, 2018) [173,174] showed that NOG1 also regulates the guard cell aperture in response to biotic and abiotic stimuli. Pant et al. (2022) [175] demonstrated that the overexpression of NOG1 conferred drought tolerance to rice.

In Manzano et al. (2020) [176], Arabidopsis mutants from NUC1 and NUC2 genes, both coding for ribosome biogenesis regulators, were submitted to two lighting regimes (red light and darkness). The results indicated that the darkness compromised the ribosome synthesis in particular. Plants exposed to red light, a ribosome synthesis activator, showed more resilience only when NUC2 function was preserved. Interestingly, the purpose of this research was to identify genetic backgrounds that can be advantageous to the plants’ adaptation to spaceflight. The plants are the key enablers of longer human missions to space due to the vital resources they can provide (biomass, revitalized atmosphere, purified water, and waste recycling) [177]. However, plant cultivation in such unique conditions (microgravity, weak magnetic field, darkness) is hardly manageable. Ground experiments such as the ones performed by Manzano et al. (2020) [176] are a useful complement to the ones that have been carried out in space.

Even though no direct evidence of ribosome heterogeneity was produced from them, the studies that applied polysome or ribosome profiling approaches are noteworthy for proving that abiotic stressors can induce differential translational landscapes [178]. The polysome profiling uses sucrose-gradient centrifugation to separate the translatome. The translated transcripts, heavier due to ribosome association, are deposited in the bottom, isolated, and then quantified [179].Through ribosome profiling, however, the measuring of the translatome is carried out in a sequencing-based manner [180]. Nucleases degrade the portions of the mRNA that are not coupled with the translation machinery, and the remaining fractions, ~30 nt translating sequences known as ribosome footprints, are sequenced [181]. Unlike polysome profiling, this methodology enables a high-resolution mapping of the mRNA segments actively recruited by ribosome that may include the identification of alternative start codons and novel open reading frames [182].

Translational activity under stressed and unstressed conditions was compared using these techniques and revealed that many abiotic stressors such as hypoxia [60,183,184], light deprivation [59], water deficit [58,185], salinity [186,187], and heat [187,188] strongly drive the selection of translating mRNA pools. In addition, some important regulatory cues such as the active translation of uORFs as a strategy to quickly adjust the translatome to environmental changes were detected by Liu et al. (2013), Juntawong et al. (2014), and Lei et al. (2015) [58,59,60] through ribosome profiling.

## 5. Ribosome Heterogeneity and Abiotic Stress Regulation in Plants: What Is Next?

Breaking the code of plant ribosome heterogeneity induced by abiotic stress is the main requisite to successfully outline ribosome engineering projects aiming to breed cultivars for superior performance under fluctuating environments. It is a continuous pursuit that should follow some guiding points:

- Manipulating the ribosomes is modifying a system:
  The role of a heterogeneous ribosome subgroup in the plant response to abiotic stress can only be fairly characterized under the prism of a whole regulatory system.
  The ribosome heterogeneity applies a translational selection on mRNA pools which are themselves the product of transcriptional regulation. Therefore, to identify which ribosome feature is worth being engineered, it is necessary not only to understand its impact on a certain translation outcome but also how it relates to the other regulation layers.
- The complexity of abiotic stressors must be addressed:
  Although they meet the goals of basic science, most of the studies limit the spectrum of plant response to abiotic stresses by analyzing each type of them individually. A single kind of stress at a time is the opposite scenario of what happens in nature. The environmental conditions are essentially unstable. The plants usually have to tackle different abiotic stressors simultaneously. Thus, their reaction in this case is surely not the same as when only one stress is applied, as demonstrated by Rasmussen et al. (2013) [189]. So, for plant breeding purposes, the investigations of ribosome heterogeneity induced by a combination of environmental stress must be intensified. This way, the ribosome engineering can be centered on data that reflect more consistently the conditions found in the field.
- Synthetic biology techniques should be optimized for plant research:
  While techniques such as CRISPR/Cas have been broadly used to precisely edit the genome of crop species and develop more sustainable cultivars [190], other ones remain to be mastered by plant research. Methods of in vitro ribosome synthesis such as the “Integrated synthesis, assembly, and translation” (iSAT) [191,192] and “In vitro ribosome synthesis and evolution” (RISE) [193] enable the production and investigation of ribosome variants. Once optimized and fully explored by plant biologists, significant advances in the knowledge of plant ribosomes can be achieved through them.

## Data Availability

Not applicable.
